# Analysis of the Vulvar Skin Microbiota in Asymptomatic Women and Patients With Vulvar Lichen Sclerosus Based on 16S rRNA Sequencing

**DOI:** 10.3389/fcell.2022.842031

**Published:** 2022-04-05

**Authors:** Xiaoxiao Liu, Yingying Zhuo, Yunlu Zhou, Jun Hu, Hongwu Wen, Changji Xiao

**Affiliations:** ^1^ Obstetrics and Gynaecology Department, Peking University First Hospital, Peking University, Beijing, China; ^2^ School of Basic Medical Sciences, Peking University, Ministry of Education, Beijing, China

**Keywords:** Vulvar lichen sclerosus, Skin microbiota, 16S rRNA, Lactobacillus, Alpha diversity, Beta diversity

## Abstract

Vulvar lichen sclerosus (VLS) is a chronic inflammatory skin disease that brings life-long and psychological distress to patients. It remains unclear whether this condition is related to changes in the skin microbial community. The aim of this study was to evaluate the compositional characteristics of the vulvar skin microbiota between VLS patients and asymptomatic postmenopausal women. We included 60 cases of postmenopausal patients in the outpatient vulvar clinic of Peking University First Hospital from August 2020 to October 2020. Thirty-one patients were diagnosed with VLS by vulvar skin biopsy (VLS group), while 29 women were asymptomatic volunteers (control group). DNA was extracted from vulvar skin swabs of the VLS and control groups. The V3-V4 fragments of 16S rRNA were targeted for high-throughput sequencing and gene sequence analysis. The sequencing results were analysed by *α* diversity, *β* diversity, species composition, LEfSe analysis to compare the compositional differences of the vulvar skin microbiota between the two groups. Our study revealed that at the phylum level, patients with VLS had a lower relative abundance of Firmicutes (*p* < 0.0001) and a higher relative abundance of Proteobacteria than the control group (*p* < 0.0001). At the genus level, *Lactobacillus* spp. accounted for the largest proportion of the microflora in the asymptomatic controls, while the proportion of *Prevotella* spp. in the VLS group was the highest. In the VLS group, the relative abundance of *Finegoldia* spp., *Ralstonia* spp., *Peptoniphilus* spp., *Anaerococcus* spp., *Campylobacter* spp., *Providencia* spp. *Kelbsiella* spp., *Ezakiella* spp., and *Escherichia-Shigella* spp. was significantly increased compared with the control group. Although there was no significant difference in the *α* diversity of the vulvar skin microbiota, the *β* diversity differed significantly between the two groups.

## Introduction

Vulvar lichen sclerosus (VLS) is a chronic inflammatory skin disease of the vulva that may lead to vulvar scarring and sexual dysfunction. VLS affects premenarchal children and postmenopausal women, with an incidence rate ranging from 1 in 70 to 1 in 1,000 (Fruchter et al., 2017). However, the actual incidence may be underestimated. The pathogenesis of VLS remains uncertain. Autoimmune factors, genetic background, hormonal factors, and local factors have been associated with the pathogenesis of VLS. With the development of microbiome sequencing technology, it has been demonstrated that changes in the composition of the human microbiota may contribute to the development of chronic inflammatory diseases. Symbiotic bacteria can beneficially regulate host immunity, reducing the risk of infection-induced autoimmune disease and/or inflammation, while maladaptive dysbiosis of the microbiome may lead to disruption of immune homeostasis and disease. It has been reported that colonization and alteration of the skin microbiota are associated with a variety of skin diseases, such as dermatitis, rosacea, and psoriasis (Holmes et al., 2013; Nakatsuji et al., 2017; Thio et al., 2018; Wang et al., 2020). Currently, microbiome profiling has been used to explore the microbial composition of the vulvar skin and the role of the microbiome in vulvar health and disease (JayaramA et al., 2014; Pagan et al., 2021; Murina et al., 2020). However, few studies have focused on the correlation between vulvar skin microbiota and VLS. The aim of our study was to evaluate the compositional characteristics of the vulvar skin microbiota between VLS patients and asymptomatic women using 16S rRNA sequencing.

## 2 Materials and Methods

### Patients and Sample Collection

Thirty-one VLS patients and 29 asymptomatic volunteers were recruited from the outpatient clinic of Peking University First Hospital from August 2020 to October 2020. The inclusion criteria for the VLS group were postmenopausal patients pathologically diagnosed with VLS by vulvar skin punch biopsy. The inclusion criteria for the control group were postmenopausal women without any vulvar symptoms or vulvar skin lesions. Women with acute vulvar infection, vaginitis, cervicitis or pelvic inflammation, use of topical glucocorticoids, oral or topical antibiotics within the last month, history of malignant tumours, radiotherapy/chemotherapy, or systemic skin diseases were excluded. Basic information (including body mass index, race and dietary habits) and medical history were recorded. The Cattaneo Clinical Score was used to evaluate the severity of VLS (Cattaneo A et al., 1991). Vaginal secretion wet pap was taken routinely to rule out bacterial vaginosis. Skin samples were collected from the labium majus and labium minus with a sterile swab in the clinic room. The sampling area was approximately 3 cm × 3 cm. Samples were stored in 2 ml sterile Eppendorf tubes at −80°C for DNA extraction.

### Sequence Processing and Data Analysis

Genomic DNA was extracted using the bacterial Genome DNA extraction kit [Hi-Swab DNA Kit, TIANGEN ^®^ BIOTECH (BEIJING) CO., LTD.]. The primer pairs used to amplify the hypervariable 16S rRNA regions V3-V4 were 341F (5′-CCTAYGGGRBGCASCAG-3′) upstream and 806R (5′-GGACTACNNGGGTATCTAAT-3′) downstream. The HiSeq 2,500 Illumina sequencing platform was used for high-throughput sequencing. High-quality sequences (length> 100 bp, mass fractions> 20) were clustered at a 97% similarity level with the Silva database into an operational taxon (OTU). The Shannon diversity index was used to compare the *α* diversity of the microbial communities between the two groups. We used the ACE and Chao1 indices to compare the richness of the microbiota and the Pielou index to compare the evenness. Richness and evenness were both measurements of *α* diversity. The Mann-Whitney test, principal coordinate analysis (PCoA) and ANOSIM were used to evaluate the differences in abundance and β diversity between the two groups. Quantitative Insights into Microbial Ecology (QIIME2) and the R package vegan were used for data processing and graph drawing.

## Results

### The Demographic Information of Vulvar Lichen Sclerosus Patients and Controls

The demographic information of the VLS patients and controls is shown in Table 1. All the patients and controls enrolled were Asian. The median Cattaneo Clinical Score of VLS patients was 8. There was no significant difference in age, allergy history, dietary habits, or daily hygiene habits between the two groups (*p* = 0.3103, 0.3022, 0.9999, and 0.7765 respectively), except for body mass index (BMI) (*p* = 0.0110).

**TABLE 1:** The demographic information of the VLS patients and controls.

	VLS (*n* = 31)		CON (*n* = 29)		*p* Value
Age, median (IQR)	61	56.5–62.5	60	57–68	0.3103
BMI, median (IQR)	23.4	22.6–25.9	21.3	19.1–24.4	0.0110*
Dietary habits	—	—	—	—	0.9999
Vegetarian diet, *n* (%)	4 (12.9)		3 (10.34)		
Normal diet, *n* (%)	27 (87.1)		26 (89.66)		
Hygiene habits Frequency of cleaning vulva	—	—	—	—	0.7765
≥3/week, *n* (%)	23 (74.19)		20 (68.97)		
<3/week, *n* (%)	8 (25.81)		9 (31.03)		
Drug allergy	—	—	—	—	0.3022
Allergy history, *n* (%)	7 (22.58)		3 (10.34)		
No history of allergies, *n* (%)	24 (77.42)		26 (89.66)		

Note: Continual variables are presented as median values (25–75th percentile), and categorical variables are presented as frequencies and proportions. Data were compared using Chi-squared, Fisher’s exact or Mann–Whitney U tests as appropriate. *p values less than 0.05 were considered statistically significant.

### Data Results and Phylogenetic Analyses (OTUs)

**FIGURE 1:**
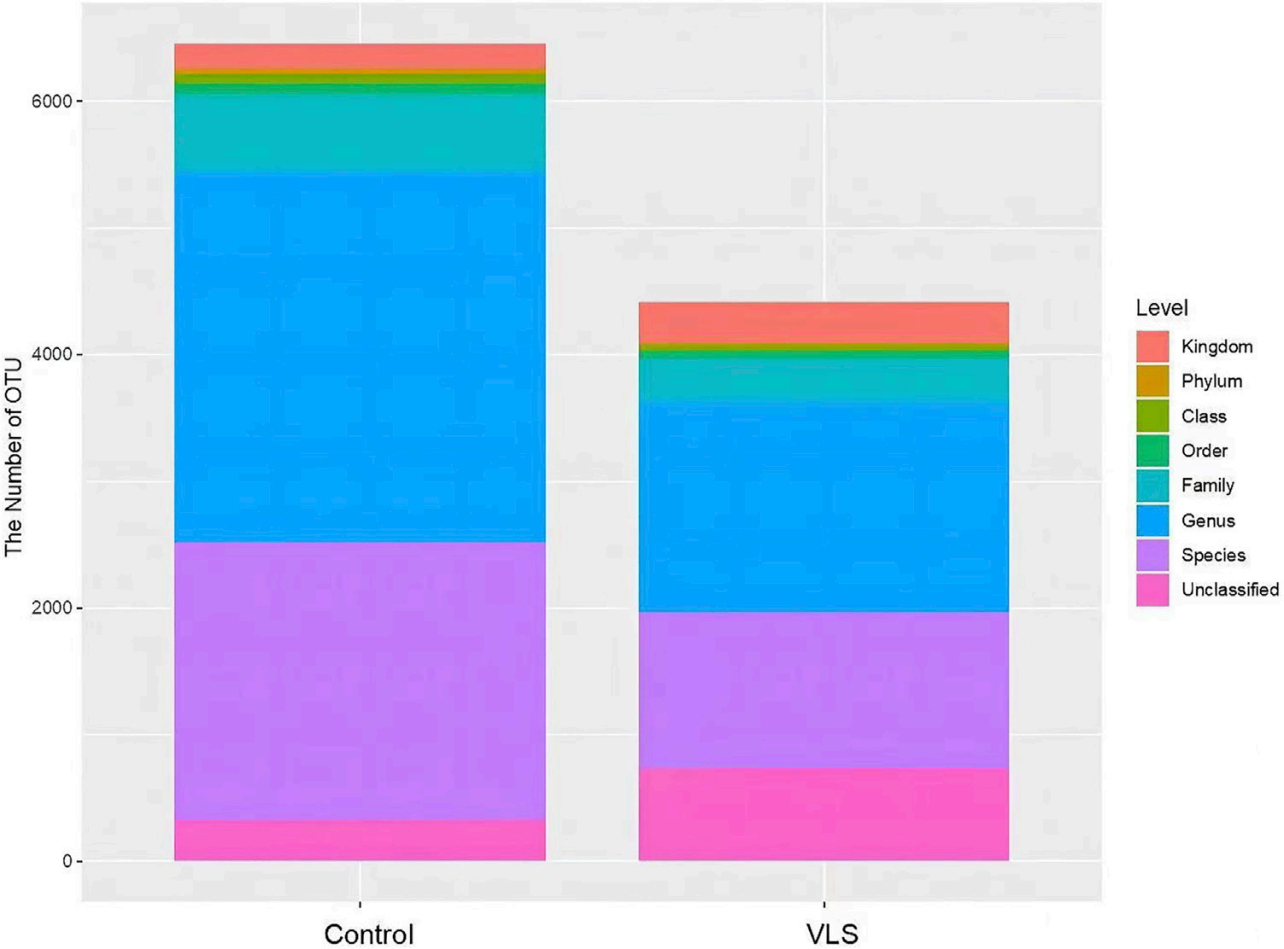
Comparison of OTU numbers at all levels between the VLS group and control group.

A total of 5,578,399 sequences were generated with an average of 92,973 sequences per sample. After quality filtering, a total of 5,537,018 high-quality sequences were obtained for downstream analysis. These sequences were divided into 10,868 OTUs, including VLS 4415 and controls 6,453. There were 679 overlapping OTUs between the two groups. Statistical data of identified OTU numbers at all levels in the VLS group and control group were shown in Table 2 and Figure 1.

**TABLE 2:** Statistical data of taxa at all levels in the VLS group and control group.

Group	Kingdom	Phylum	Class	Order	Family	Genus	Species	Unclassified
Control (OTUs)	198	41	75	85	613	2,923	2,191	327
VLS (OTUs)	318	20	46	61	339	1,666	1,227	738

### Changes in the Structure of the Vulvar Microbiota in Patients With Vulvar Lichen Sclerosus

**FIGURE 2:**
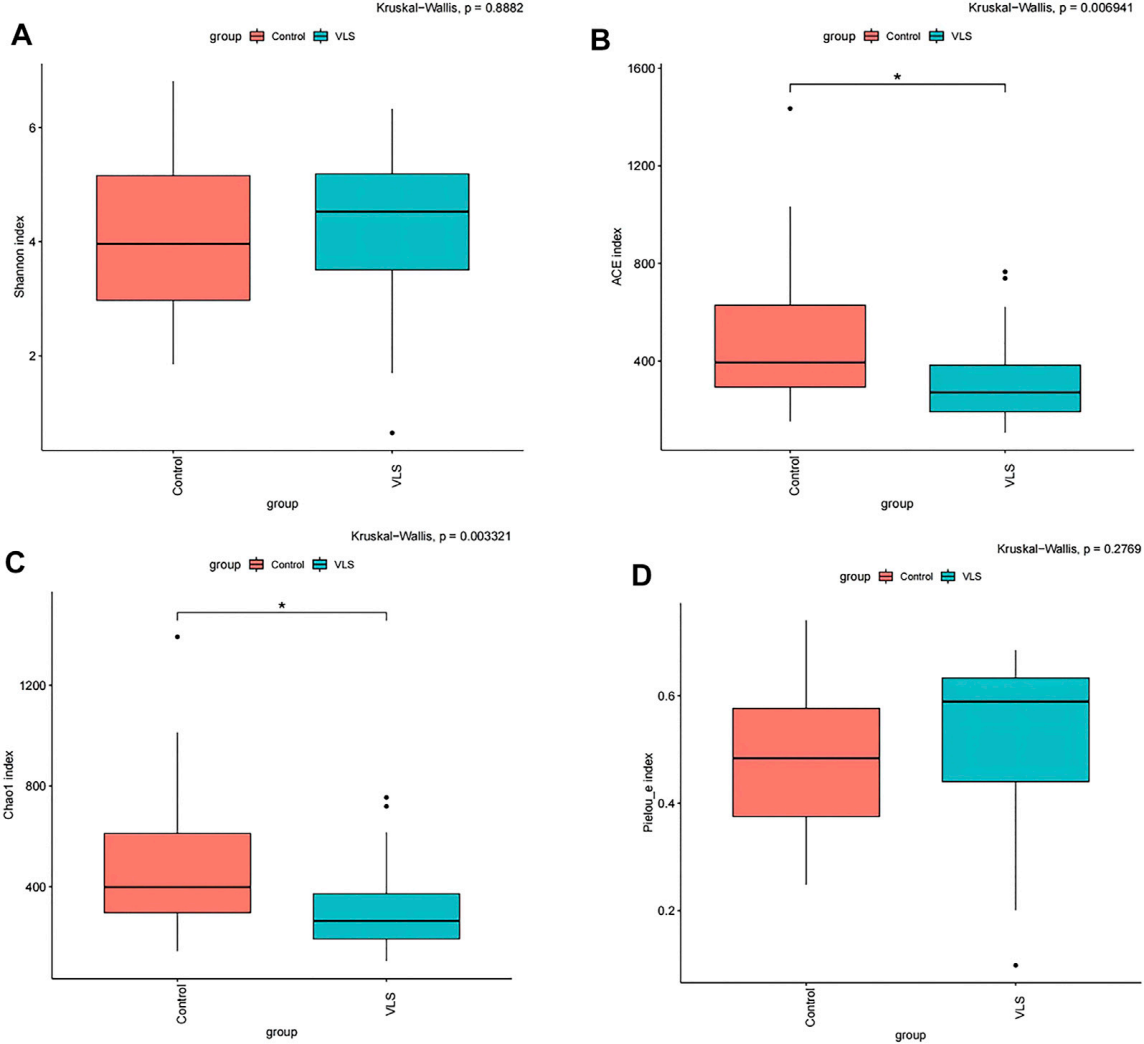
**(A)** Shannon diversity index (*α*-diversity) of the vulvar microbiota between the VLS and control groups. ACE index **(B)** and Chao1 index **(C)** (richness) of vulvar microbiota between the VLS and control groups. **(D)** Pielou index **(D)** (evenness) of vulvar microbiota between the VLS and control groups. *p values less than 0.05 were considered statistically significant.

**FIGURE 3:**
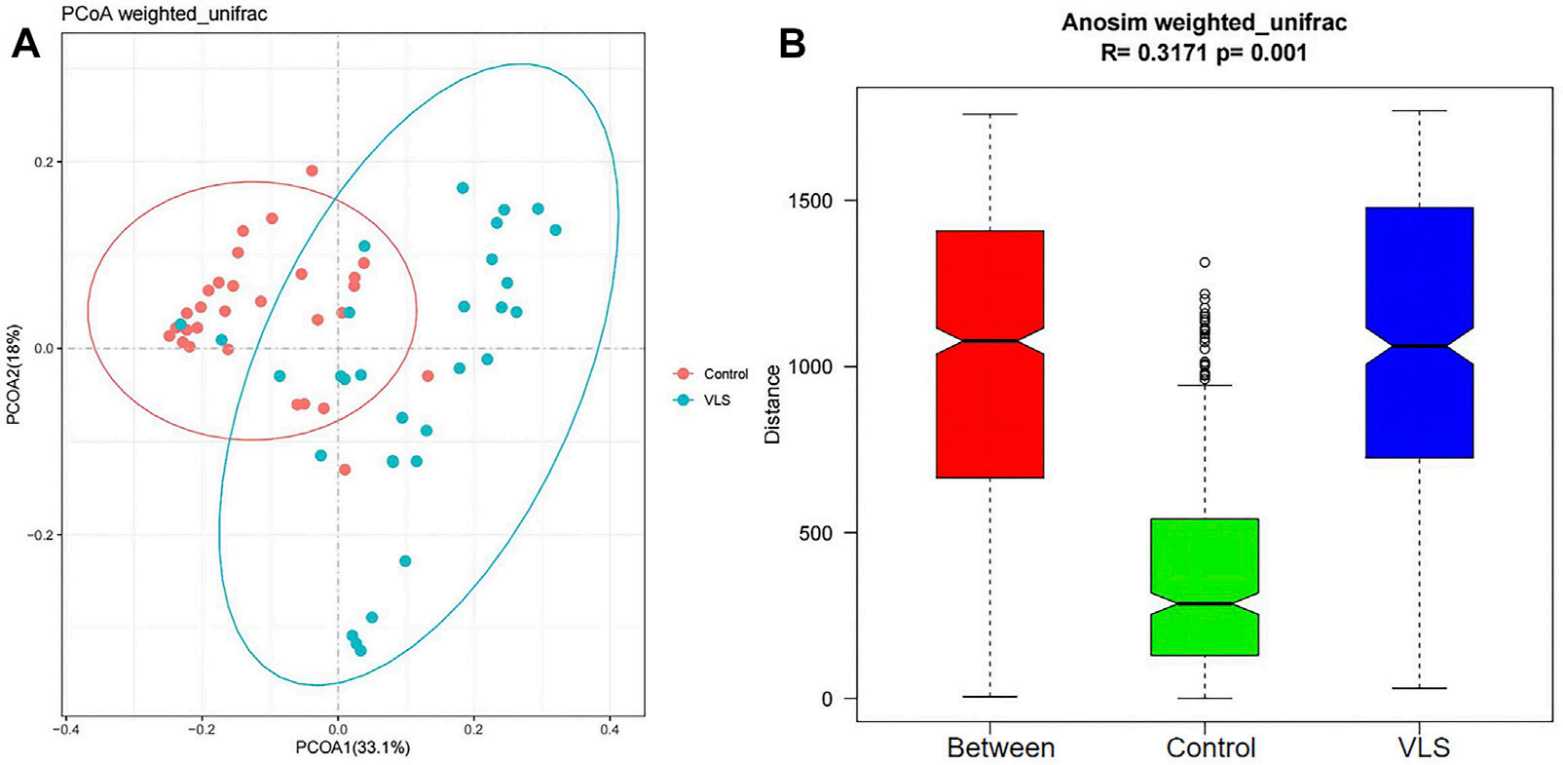
**(A)** The results of principal coordinate analysis (PCoA) based on weighted UniFrac showed that the bacterial microbiome clustered separately in the VLS and control groups. **(B)** The plot of ANOSIM analysis measured the β diversity of vulvar microbiota between the VLS and control groups. (The Between column is the merged information of the two groups.)

We found that there was no difference in the *α* diversity as measured by Shannon’s index (M-W test: *p* = 0.8882) (Figure 2A). The ACE and Chao1 richness indices in the VLS group were significantly lower than those in the control group (M-W test: *p* = 0.0069, *p* = 0.0033) (Figures 2B,C). The Pielou index for measurements of evenness was not significantly different between the two groups (M-W test: *p* = 0.2769) (Figure 2D). In terms of *β* diversity, which was used to compare the composition of taxa in the two cohorts, weighted UniFrac PCoA distinguished the VLS group from the asymptomatic control group (Figure 3A). The ANOSIM analysis showed R > 0, *p* = 0.001, indicating significant differences between the two groups (Figure 3B).

### Study on Changes in the Vulvar Microbiota in Vulvar Lichen Sclerosus Patients

**FIGURE 4:**
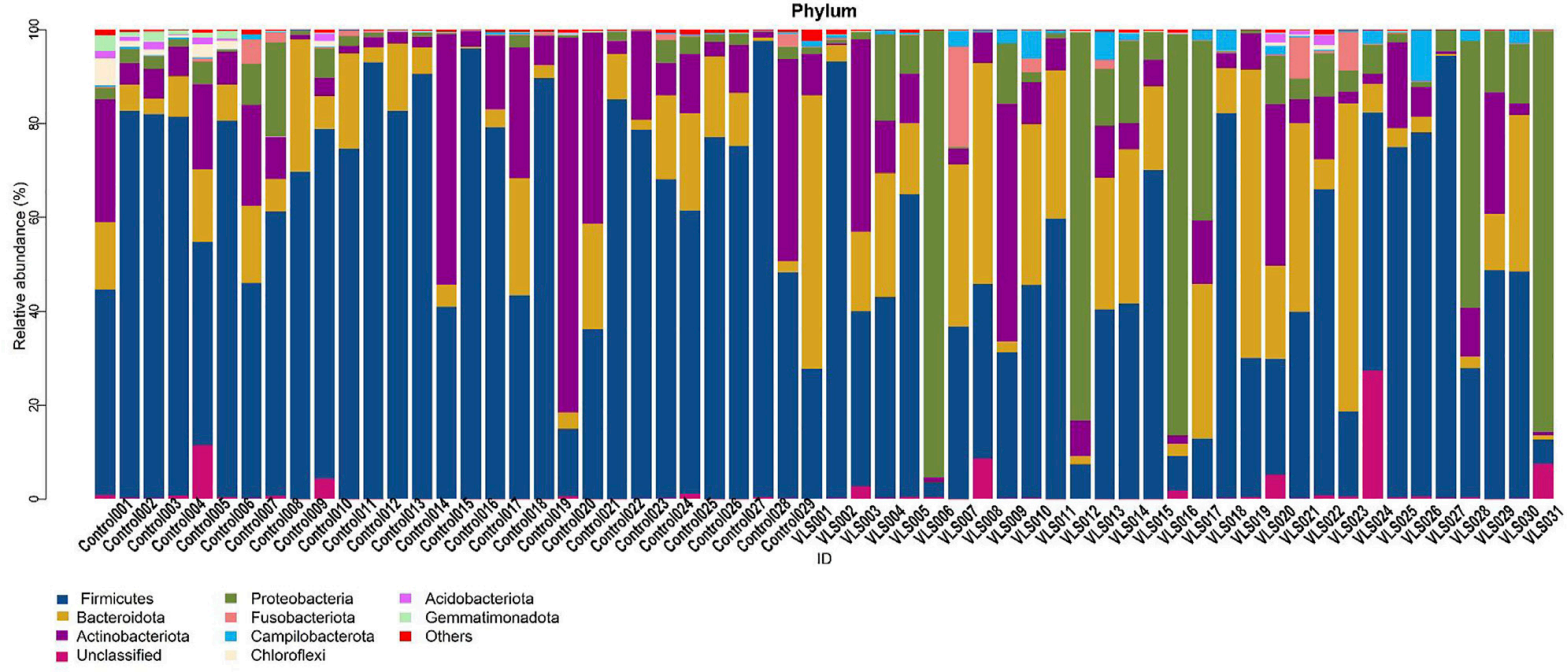
Species’ relative abundance map at the phylum level of the VLS and control groups.

**FIGURE 5:**
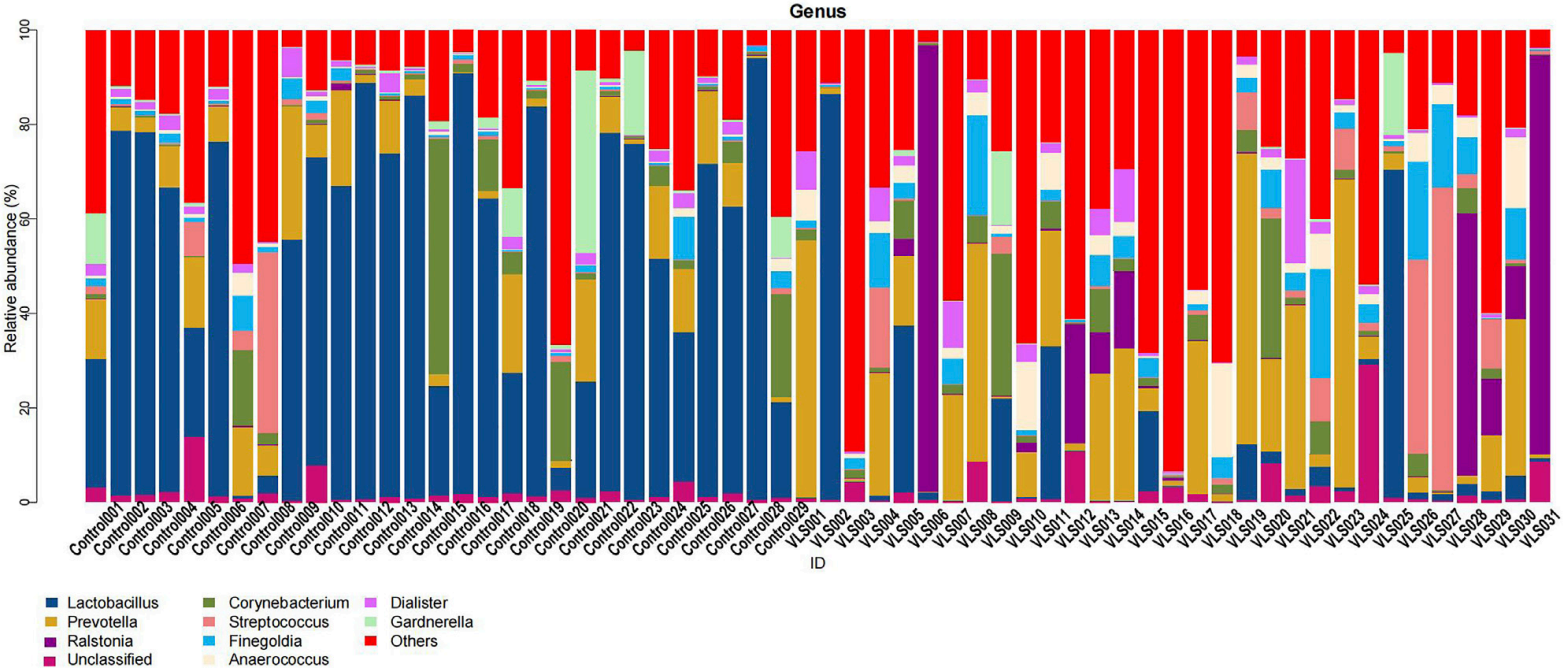
Species’ relative abundance map at the genus level of the VLS and control groups.

**FIGURE 6:**
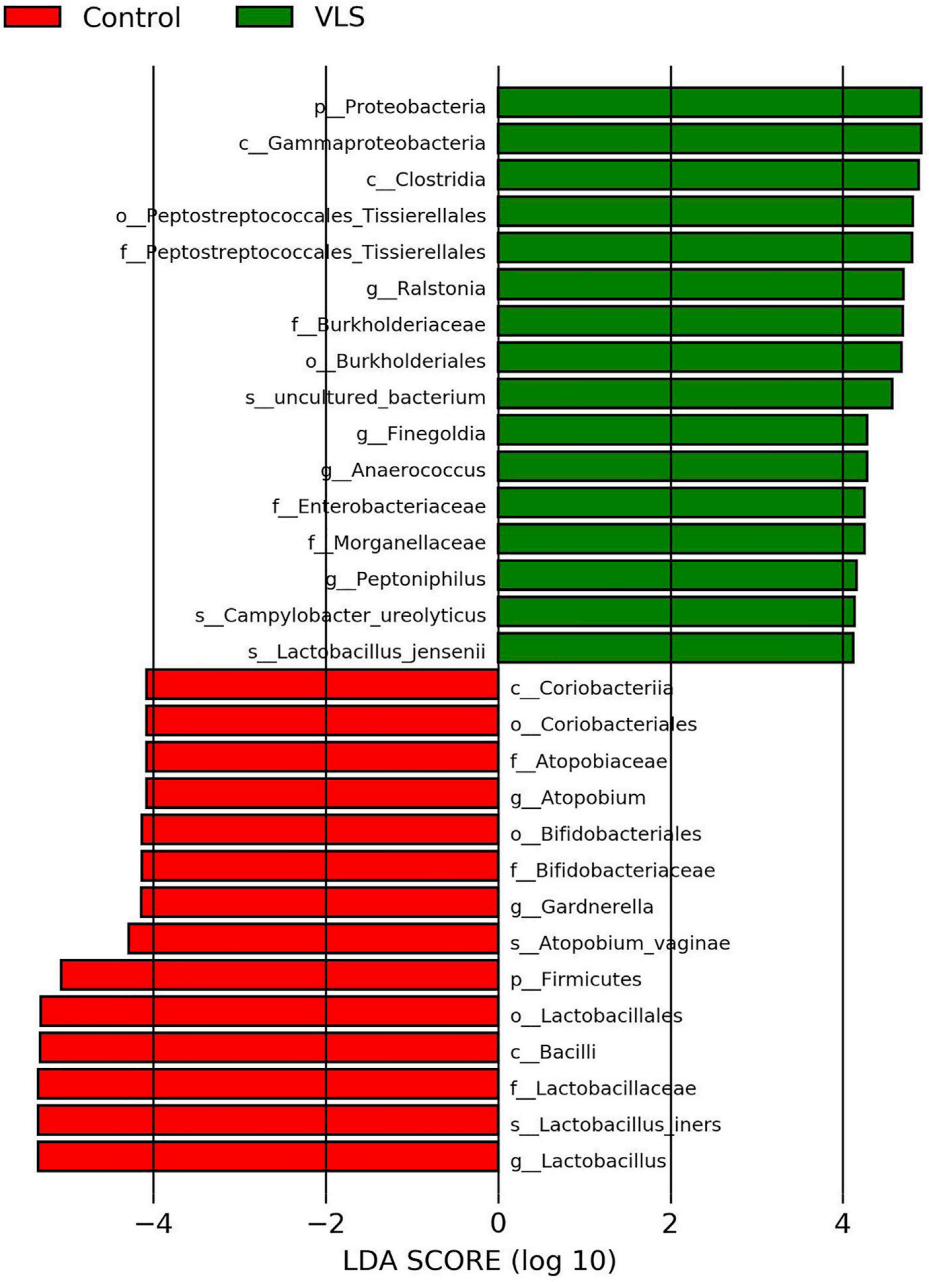
LEfSe analysis of the VLS group and control group (LDA >4.0).

According to the Silva analysis, the samples were divided into 45 phyla and 752 genera. The distribution of phyla and genera indicated differences between VLS patients and controls. The four most common phyla in both asymptomatic controls and VLS skin were Firmicutes, Bacteroidetes, Actinobacteria and Proteobacteria (Figure 4). Compared with asymptomatic controls, patients with VLS had an increased relative abundance of Proteobacteria (VLS vs. control, 19.22 vs. 2.66%, *p* < 0.0001) and a decreased relative abundance of Firmicutes (VLS vs. control, 43.47 vs. 68.67%, *p* < 0.0001) (Table 3). At the genus level, 9 key functional bacterial genera (*Finegoldia* spp., *Ralstonia* spp., *Peptoniphilus* spp., *Anaerococcus* spp., *Campylobacter* spp., *Providencia* spp. *Kelbsiella* spp., *Ezakiella* spp., and *Escherichia-Shigella* spp.) increased significantly between the VLS group and the control group; however, only 3 genera (*Lactobacillus* spp., *Atopobium* spp. and *Gardnerella* spp.) decreased significantly in VLS patients. *Lactobacillus* was predominant in the control group, and its relative abundance was significantly decreased in the VLS patients (54.17 vs. 9.73%, *p* < 0.0001). *Prevotella* spp. (belonging to Bacteroidetes) was predominant in the VLS, but there was no significant difference when compared with the control group (17.69 vs. 8.96%, *p* = 0.3630), which was consistent with the Bacteroidetes phyla (Table 4). Species’ relative abundance at the genus level of the VLS and control groups were shown in Figure 5. We also used the LEfSe algorithm to identify the specific taxa that were variably distributed in the groups. By using the default LDA cut-off of+/−4.0, we found that 16 taxa were overrepresented and 14 taxa were underrepresented in the VLS group compared to the control group. The key categories with significant differences between the VLS group and the control group are shown in Figure 6.

**TABLE 3:** The mean relative abundance of microbiota at phylum level in the VLS group and control group.

	CON (%)	VLS (%)	*p* Value
Firmicutes	68.67	43.47	*p* < 0.0001 *
Bacteroidetes	10.42	21.03	0.1021
Actinobacteria	15.09	10.55	0.5009
Proteobacteria	2.66	19.22	*p* < 0.0001 *
Campilobacterota	0.24	1.75	0.004
Fusobacteriota	0.53	1.49	0.327
Others#	2.39	2.49	NA

Note: Mann-Whitney test was used to compare the differences between the VLS, and control groups. *p values less than 0.05 were considered statistically significant. # Others include the taxa that cannot be classified and taxa with relative abundance of less than 1%. NA: not applicable.

**TABLE 4:** The mean relative abundance of the microbiota at genus level in the VLS group and control group.

	CON (%)	VLS (%)	*p* Value
Finegoldia spp.	1.65	5.66	0.0032*
Ralstonia spp.	0.08	10.21	*p* < 0.0001*
Peptoniphilus spp.	0.84	3.37	*p* < 0.0001*
Anaerococcus spp.	0.62	4.01	0.0001*
Campylobacter spp.	0.20	1.74	0.0008*
Providencia spp.	0	2.97	0.0010*
Kelbsiella spp.	0.01	1.34	0.0016*
Ezakiella spp.	0.07	1.02	0.0025*
Escherichia-Shigella spp.	0.29	2.49	0.0046*
*Lactobacillus* spp.	54.17	9.73	*p* < 0.0001*
Atopobium spp.	2.23	0.40	0.0002*
Gardnerella spp.	3.43	1.21	0.0001*
Prevotella spp.	8.96	17.69	0.3630
Staphylococcus spp.	1.92	1.24	0.0700
Streptococcus spp.	2.13	5.72	0.1955
Corynebacterium spp.	5.23	4.53	0.2223
Dialister spp.	1.59	2.95	0.8417
Porphyromonas spp.	0.15	1.88	0.0728
Actinomyces spp.	0.47	2.06	0.08020
Faecalibacterium spp.	0.08	1.03	0.1850
Fenollaria spp.	0.24	1.03	0.2195
Bacteroides spp.	0.18	1.21	0.8412
Veillonella spp.	0.55	1.82	0.8879
Others#	14.91	14.69	NA

Note: Mann-Whitney test was used to compare the differences between the VLS, and control groups. *p values less than 0.05 were considered statistically significant. # Others include the taxa that cannot be classified and taxa with relative abundance of less than 1%. NA: not applicable.

## Discussion

Skin microecology is a skin microbiota composed of millions of bacteria, fungi, and viruses (Byrd et al., 2018). Like the microbes in the gut, skin microbes play a crucial role in the regulation of the immune system. The composition of the microbial community depended on the physiological state of the skin, and the relative abundance of bacteria changed with different humidity and sebaceous microenvironments. The vulva microbiota is diverse, and the normal vulva microbiota includes the characteristics of the vagina, urethra, and colon (Brown et al., 2007; Pagan et al., 2021). Previous studies on the vulvar skin microbiota, from cultivation to culture-independent molecular techniques, have shown that *Lactobacillus and Corynebacterium* spp., as well as *Staphylococcus aureus* and Prevotella, were the main bacterial components of healthy vulvar skin. (Elsner and Maibach, 1990a; Costello et al., 2009; Bruning et al., 2020). Wolf et al. (2017) transplanted vulvar skin of VLS patients onto the thigh and returned to normal skin, while normal skin was transplanted onto the vulvar area of VLS patients, and the skin turned into VLS. This suggested that the local microenvironment could be associated with the pathogenesis of VLS. Therefore, the natural microbiome of the vulvar skin may play a significant role in immunizing and maintaining vulva health, as well as preventing invasive organisms from gaining a foothold.

*Lactobacillus* is the permanent probiotic of the vaginal mucosa, which constitutes a majority of the vaginal flora. As the portal of the female reproductive tract, the flora composition of the vulva is influenced by the flora of the vagina. Therefore, lactobacilli dominate the vulva flora of healthy women (Brown et al., 2007; Costello et al., 2009; Murina et al., 2020). Researchers have used 16S rRNA sequencing to study the vulvar flora in premenopausal nonpregnant women. Samples were taken from 10 Japanese women before and on the second day of menstruation. *Lactobacillus* was dominant in all 7 subjects before menstruation and during the menstruation period (Shiraishi et al., 2011). In our study, we also found that the relative abundance of *Lactobacillus* in the asymptomatic control group was 54.17%, while the proportion of other bacterial groups was small. However, in the study of Chattopadhyay et al. (2021), sequencing and analysis of the vulva skin flora of 5 VLS child patients and 3 healthy children found that at the genus level, *Porphyromonas* spp., *Prevotella*, *Corynebacterium* and *Peptoniphilus* were dominant in the two groups, which may be due to the difference in vulva flora between women and children.

There was limited evidence on the skin flora of VLS patients. Watchorn et al. found a significant increase in the relative abundance of *Prevotella* compared to the normal control group in a study of male sclerosing lichens (20 vs. 4%) (Watchorn et al., 2021). Chattopahyay et al. (2021) found that the relative abundance of *Prevotella* in the vulvar flora of VLS children was significantly higher than that of healthy children. However, we found that there was no significant difference in the relative abundance of *Prevotella* between the VLS group and the control group (17.69 vs. 8.96%, *p* = 0.3630), which indicated that the vulvar skin microbiota of VLS patients might vary with age and postmenopausal status.

In addition to *Prevotella*, the abundance of other anaerobes in the vulva skin flora of VLS patients was also higher than that of healthy women. Guet-Revillet et al. (2014) found that strict anaerobes, such as *Prevotella*, *Peptonophilus*, and *Porphyromonas gingivalis,* were present in 87% of chronic suppurative bacteria, suggesting that these bacteria may be involved in chronic inflammation. In the same way, these anaerobic bacteria may also be involved in the formation of chronic skin inflammation and the development of VLS through similar immune regulation. Our study found that Firmicutes, Bacteroidetes, Actinobacteria, and Proteobacteria accounted for more than 97% of the total sequence in the vulvar skin swab samples in both groups. Compared to the control group, the number of Proteobacteria in the VLS group was significantly increased (19.22 vs. 2.66% *p* = 0.000997). Previous studies have shown that *Corynebacterium* predominate on wet skin, while the predominant colonization mixture is Proteobacteria and Flavobacteria on dry skin (Grice et al., 2009). Our result is consistent with the dry vulvar skin condition in patients with VLS.

Other potential factors influencing the vulvar skin microbiota include menstrual cycle, body mass index, pH, temperature, dryness, ethnicity, dietary habits, antibiotic use, and other factors, which play a crucial role in maintaining and stabilizing the microbiota. Vongsa et al. (2019) found that Lactobacillus spp. were more prevalent on the vulva of women with normal BMI, whereas Corynebacterium spp. and Anaerococcus spp. were more prevalent on the vulva of women with a high BMI. Therefore, it should be noted that our results may be influenced by BMI, as there were significant differences in BMI between the VLS patients and asymptomatic controls. It has been speculated that the vulva pH value may be somewhere between the skin value (estimated at pH 4.7) and the vagina value (average pH 3.5), which ranges from 3.8 to 4.2 in the menstrual cycle (Chen et al., 2017). A range of factors may affect the vulva pH value, including endogenous factors (e.g., humidity, sweat and vaginal discharge, menstrual cycle, urine and faecal pollution, anatomical folds, genetics, and age) and exogenous factors (such as soap, detergent, cosmetics, lubricants and spermicide, with tight clothes or sanitary napkin shade, shaving, and leather products). Long-term dryness of vulvar skin can significantly reduce its pH (Elsner and Maibach, 1990b), and pH can subsequently influence the growth of various microorganisms.

Therefore, our study has the following shortcomings. Due to the limitations of 16S rRNA, this study was not able to assess whether vulvar skin fungi and viruses changed in VLS patients. Second, questions have been raised regarding whether skin biopsy samples of the deep epidermis, dermis and glands might provide additional information on the skin microbiota (Grice et al., 2009). Therefore, swab sampling may have limitations in the research results. Finally, due to the limitations of the algorithm, our evaluation could not reach the species level, and metagenomics is required for further analysis in future work.

## Conclusion

This study compared the compositional characteristics of the vulvar skin microbiota in patients with VLS and asymptomatic menopausal women. Although there was no significant difference in the *α* diversity, the *β* diversity differed significantly between the two groups. The predominant bacteria on the vulvar skin of asymptomatic women were *Lactobacillus* spp., while the proportion of *Prevotella* spp. was most prevalent in the VLS group. The relative abundance of *Finegoldia* spp., *Ralstonia* spp., *Peptoniphilus* spp., *Anaerococcus* spp., *Campylobacter* spp., *Providencia* spp. *Kelbsiella* spp., *Ezakiella* spp., and *Escherichia-Shigella* spp. was significantly increased in the VLS group. Metabolomics and transcriptomics are required to explain the relationship more completely between VLS and the vulvar skin microflora, which may provide a theoretical basis for clarifying the pathogenesis of VLS and new concepts for its treatment.

## Data Availability

The datasets presented in this study can be found in online repositories. The names of the repository/repositories and accession number(s) can be found below: https://www.ncbi.nlm.nih.gov/, PRJNA791515.
